# High burden of *Schistosoma mansoni* infection in school-aged children in Marolambo District, Madagascar

**DOI:** 10.1186/s13071-017-2249-7

**Published:** 2017-06-24

**Authors:** Stephen A. Spencer, James M. St. John Penney, Hannah J. Russell, Anthony P. Howe, Cortland Linder, Andriamahitsisambatra L. D. Rakotomampianina, Anjara M. Nandimbiniaina, S Bertel Squire, J. Russell Stothard, Amaya L. Bustinduy, Alain M. Rahetilahy

**Affiliations:** 1 Royal United Hospitals Bath NHS Foundation Trust, Bath, UK; 2 The University of Manchester Medical School, Manchester, UK; 3 Université d’Antananarivo, Antananarivo, Madagascar; 4 Liverpool School of Tropical Medicine, Liverpool, UK; 5 London School of Hygiene & Tropical Medicine, London, UK; 6 Ministère de la Santé Publique, Antananarivo, Madagascar

**Keywords:** Schistosomiasis, *Schistosoma mansoni*, Neglected diseases, Child health, Rural health, Madagascar

## Abstract

**Background:**

A school-based survey was undertaken to assess prevalence and infection intensity of schistosomiasis in school-aged children in the Marolambo District of Madagascar.

**Methods:**

School-aged children from six purposively selected schools were tested for *Schistosoma haematobium* by urine filtration and *Schistosoma mansoni* using circulating cathodic antigen (CCA) and Kato-Katz stool analysis. The investigators did not address soil-transmitted helminths (STH) in this study.

**Results:**

Of 399 school-aged children screened, 93.7% were infected with *S. mansoni* based on CCA analysis. Kato-Katz analysis of stool revealed *S. mansoni* infection in 73.6% (215/ 292). Heavy infections (> 400 eggs per gram) were common (32.1%; 69/ 215), with a mean of 482 eggs per gram of stool. Moderate infection intensities were detected in 31.2% (67/ 215) and light infection intensities in 36.7% (79/ 215) of infected participants. No infection with *S. haematobium* was detected by urine filtration.

**Conclusions:**

Intestinal schistosomiasis appears a considerable public health issue in this remote area of Madagascar where there is a pressing need for mass drug administration.

## Background

Schistosomiasis is common in sub-Saharan Africa and is known to be widespread in Madagascar where there is a substantial burden of disease [1, 2]. There are two endemic species of *Schistosoma*, with a geographical distribution tracking the underlying range of their permissive snail hosts [3, 4]. *Schistosoma mansoni* causes intestinal schistosomiasis and is found in eastern and southern areas of Madagascar while *S. haematobium*, which causes urogenital schistosomiasis, is present in northern and western locations, with areas of co-endemicity in four regions in north-central and south-western parts of the country [5, 6]. National schistosomiasis mapping led by the Madagascar Ministry of Health demonstrated that 107 of 114 districts in Madagascar are endemic with schistosomiasis [6].

Schistosomiasis frequently leads to nonspecific effects such as anaemia, undernutrition, decreased physical fitness, impaired cognition and quality of life [7–10]; measuring these is crucial in monitoring disease morbidity [11]. *Schistosoma mansoni* in particular results in intestinal and hepatosplenic disease causing abdominal pain, blood in stool, reduced appetite and diarrhoea [12, 13]. In chronic disease states, hepatic fibrosis can develop 5–15 years after initial infection, though earlier cases have been documented in preschool children [14, 15]. This may lead to portal hypertension which can precede hepatosplenomegaly, ascites and gastro-oesophageal bleeds that can be fatal if bleeding is uncontrolled [16]. In areas with intermittent access to treatment, such as Madagascar, re-infection occurs rapidly and results in chronic schistosomiasis.

*Schistosoma haematobium* causes urogenital schistosomiasis, which often presents with haematuria. Other presenting symptoms may include urinary frequency and dysuria. Chronic infection results in fibrosis or calcification of the bladder, which may cause obstruction of the urinary tract leading to hydroureter or hydronephrosis. Treatment can cure bladder lesions and obstructive disease; however, without treatment chronic urinary schistosomiasis is associated with development of squamous cell carcinoma of the bladder. Female genital schistosomiasis (FGS) is a result of *S. haematobium* causing localised inflammation in the female genital tract. Symptoms may include dyspareunia, stress incontinence and infertility. Sandy patches in the lower genital tract are pathognomonic and are seen in association with altered vasculature. Subsequent contact bleeding increases susceptibility to HIV transmission in women with FGS [11, 17].

Preventative chemotherapy (PC) is an important public health measure entailing the delivery of medications to endemic populations to reduce morbidity and transmission rates of human helminth infections. Current schistosomiasis control activities focus on PC in the form of mass drug administration (MDA) with praziquantel (PZQ). Where the prevalence of infected school-aged children (SAC) is greater than 50%, the World Health Organization (WHO) recommends annual treatment to both SAC and adults in high risk communities. In moderate risk areas, where the prevalence amongst SAC is between 10 and 50%, MDA should be targeted towards all SAC every two years as well as to adults considered to have a higher risk of infection (such as fishermen, farmers and irrigation workers) and to pregnant or lactating women. SAC who live in regions with <10% prevalence should be treated twice during their primary schooling age [18, 19]. Using PC, the aim is control or elimination of schistosomiasis, as well as other neglected tropical diseases (NTDs) such as soil-transmitted helminthiasis (STH), onchocerciasis and lymphatic filariasis [20, 21].

There are Ministry of Health (MOH)-led NTD control programmes in 36 out of 107 affected districts in Madagascar [20]. Prohibitive costs and logistical difficulties prevent access to some districts. In more remote areas the majority of roads and tracks are often unusable during wet seasons and travel between villages is limited to foot or boat [22]. In 2015 only 1.7 million (43.6%) of the 3.8 million target population (SAC) in Madagascar received PZQ [23]. In a recent WHO publication, the coverage of PC to SAC for the African region was reported to be 41.2%, and the MOH-led PC in Madagascar is therefore above the regional average [24]. However, the annual need for PC varies between districts according to their prevalence, therefore a national approach should be guided by the differing needs for PC throughout the country. A recent paper reported on very high prevalence rates and infection intensities of both urogenital and intestinal schistosomiasis in areas of western Madagascar left untreated for over five years [25], indicating the need for urgent up-scaling of national control initiatives to 100% of the districts requiring PZQ*.* There have also been detailed publications on high schistosomiasis prevalence rates from the central highlands and western Madagascar [4, 26, 27].

The aim of this study was to investigate the presence, prevalence rates and infection intensities of *S. haematobium* and *S. mansoni* among SAC in the East of Madagascar. Here we report on a cross-sectional survey in six villages within the Marolambo District of the Atsinanana Region. This information highlights high-risk transmission areas in which communities require urgent control initiatives and provides the baseline information that is necessary to monitor the impact of intervention programmes.

## Methods

### Study area and population

**Fig. 1:**
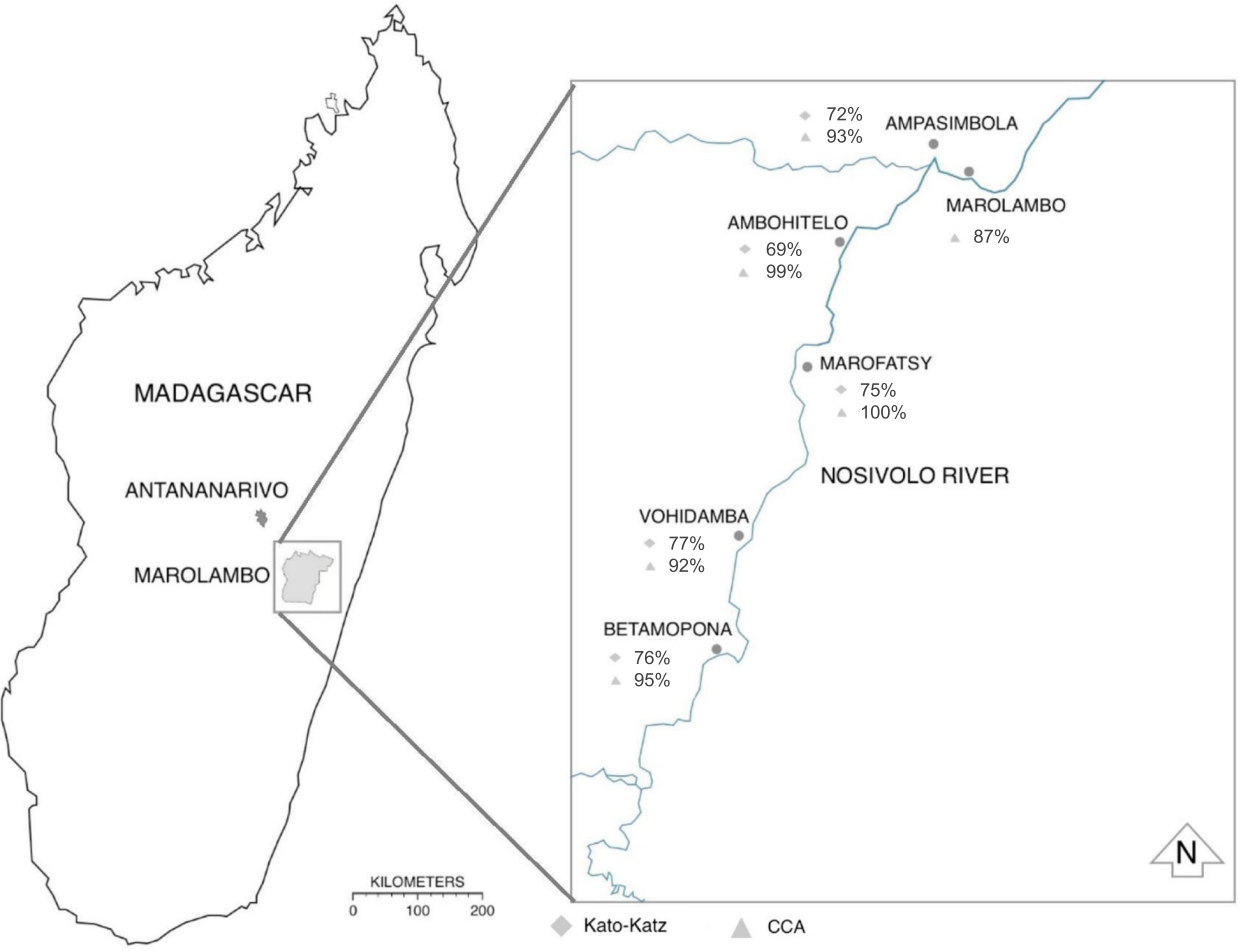
Sketch map of Marolambo showing locations of the villages screened alongside the Nosivolo River. Prevalence rates according to the CCA and Kato-Katz diagnostic tests are labelled alongside each village

This work was conducted during June-July 2015 in collaboration with the MOH, Antananarivo, Madagascar. Six villages in the Marolambo District were selected due to their accessibility and their community links with our collaborators, Durrell Wildlife Conservation Trust and MOH staff. These villages lie next to the Nosivolo River (Fig. 1), their main water source, and had not received MDA since 2008.

### Study design

Children aged 5–14 were selected from schools in six villages. Urine samples were obtained from 399 children and faecal samples from 292 children, which was the maximum number of children we could test within our fieldwork timeframe. Using school registers provided by the headteachers, an equal number of boys and girls were selected at random from each year group. This ensured an even spread in age range throughout the sample frame and equal gender representation. The numbers of valid urine and faecal samples obtained from each village were: Ampasimbola (67 urine samples; 62 faecal samples), Ambohitelo (68; 69), Marofatsy (60; 57), Marolambo (84; 0), Vohidamba (59; 53) and Betampona (61; 51).

### Parasitology methods

The day before screening all children were provided with a single stool sample container and assigned an identification number, which was recorded with their age and gender. Researchers instructed study participants on how to collect their morning stool. The next day, samples were collected by researchers. At 10 am, children were provided with a single urine sample container labelled with the same identification number and were instructed to collect a mid-stream urine sample. Children returned samples promptly before 12 pm midday. Stool and urine samples were brought to the field laboratory and processed by researchers on the same day. For the detection of *S. mansoni*, a urine-cassette assay was used to detect circulating cathodic antigen (CCA; Rapid Medical Diagnostics Tests, Pretoria, South Africa). Technicians were trained in Antananarivo in the use of CCA tests following manufacturer’s instructions and all results were verified by at least two technicians to ensure homogeneity. CCA test band reaction intensity was recorded as negative (−), trace positive (tr), single positive (+), double positive (++), or triple positive (+++) in line with previous studies [28]. Results were analysed in a binary fashion as negative or positive (including all positive recordings: tr; +; ++; +++). Urine samples were only included in the study when they were of sufficient quantity to assess using CCA.

To assess *S. mansoni* infection intensity, a single Kato-Katz thick smear containing 41.7 mg of faeces was prepared from each sample (Kato-Katz kit, Vestergaard-Frandsen, Lausanne, Switzerland). Slides were examined for *S. mansoni* eggs and any present were counted. This value was used to calculate the total number of eggs per gram (epg). Intensity of infection was classified by epg as light (1–99 epg), moderate (100–399 epg) or heavy (≥ 400 epg) [18]. Stool samples were only eligible for inclusion when they were of sufficient size to prepare a single thick smear.

For the detection of *S. haematobium*, urine samples were first tested for microhaematuria using urine-reagent strips, then 10 ml of each sample was filtered using a 12-μm polycarbonate filter (Sterlitech Corporation, Kent, USA) and examined using light microscopy to look for eggs. Only urine samples of at least 10 ml in volume were included in the study.

Slides were prepared and examined in the field. This was carried out by the four UK-based team members (three medical students and one medical doctor) following training from the University of Manchester Immunology Department and the Liverpool School of Tropical Medicine. For both *S. mansoni* and *S. haematobium*, every tenth slide was re-examined by a second team member and if a difference was identified a consensus reached. The investigators did not address STH in this study.

### Statistical analyses

Results were recorded on paper in the field and later entered into Microsoft Excel (Redmond, WA, US). Statistical analysis was performed on Stata 13 (Stata Statistical Software: Release 13. College Station, TX: StataCorp LP).

Bivariate analysis was performed to explore CCA results and infection intensity with age, controlling for gender and village. The odds ratio, 95% confidence interval (CI) and *P* were calculated for each model.

CCA results were compared to age using binary logistic regression. Intensity of infection according to epg (negative: 0, trace and single positive: 1, double positive: 2 and triple positive: 3) was simplified into 3 binary scales with outcomes of < 0, < 1, and < 2 and a pooled odds ratio for age calculated using ordinal logistic regression. Results were considered significant if *P <* 0.05.

Due to the highly skewed distribution of epg, a Spearman’s rank correlation was used to test associations between age and infection intensity.

### Consent and permissions

National and local health, educational and administrative authorities were comprehensively informed of the study. Before the start of the study in each village, open public meetings were carried out in the Malagasy language followed by question and answer sessions with the investigators. Participation information sheets and consent forms were translated into Malagasy detailing all aspects of the methodology and read to each participant in private by head-teachers, local health workers, and Malagasy investigators. The children then had the option to stamp with their fingerprint to consent to the study. It was explained that any child had the chance to withdraw from the study at any point, without any consequence.

## Results

**Fig. 2:**
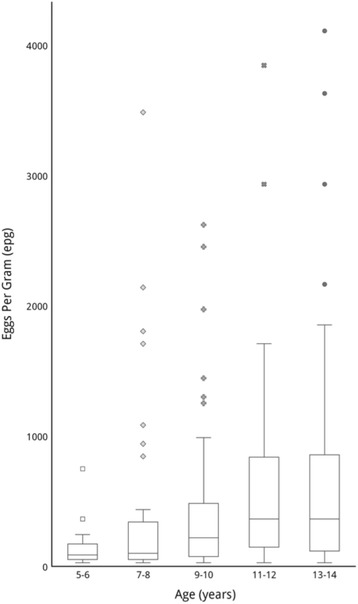
Schistosomiasis infection rates against the age groups tested in this study. This figure demonstrates the increasing parasite load with age (Spearman’s rank correlation coefficient 0.21, *P* < 0.05)

In total 399 children aged 5–14 were sampled. Of faecal samples, 215/292 (73.6%) were positive for *S. mansoni* by Kato-Katz analysis (Table 1). Light infection intensity was observed in 79/215 (36.7%) of infected participants, moderate intensity in 67/215 (31.2%) and heavy intensity in 69/215 (32.1%). Mean infection intensity across the study cohort was 482 epg (range 24–4104 epg), with no significant variation between genders. There was a non-significant variation in prevalence by village (Fig. 1). There was a positive correlation between *S. mansoni* epg and age (Spearman’s rank correlation coefficient 0.21; *P* < 0.05; Fig. 2), with an odds ratio of 1.18 for higher epg with age (95% CI: 1.11–1.26). Multivariable analysis adjusted for village and gender is shown in Table 2. No data were recorded for quality control.

**Table 1:** Characteristics and results of study by age

Study characteristic		Age (yrs)				
		5–6	7–8	9–10	11–12	13–14
Number of participants		64	85	85	79	83
Percentage female		53.13	51.76	50.59	51.90	46.99
Schistosomiasis prevalence (%)	Kato-Katz	76.56	80.00	87.06	82.28	84.34
	CCA	85.94	91.76	98.82	97.47	97.59
Sm *+* mean intensity (epg) with ranges		138.5 (24–744)	394 (24–3480)	528.9 (24–2616)	434.7 (24–3840)	732.3 (24–4104)
Sm *+* mean intensity (epg) per village	Ampasimbola	108	516	473.5	757.3	1095.3
	Ambohitelo	256	160	387.4	460.4	495
	Marofatsy	96	664	192	693	656
	Vohidamba	72	223.2	402.7	929	604.4
	Betampona	144	487	691	384	366
Sm *+* mean intensity (epg) per gender	Male	136	392.64	411.72	754.32	502.56
	Female	147.6	362.64	498.32	850.08	732.8

*Abbreviations*: *CCA* circulating cathodic antigen, *epg* eggs per gram, *Kato-Katz* microscopy using the Kato-Katz technique

**Table 2:** Multivariable analysis of schistosomiasis prevalence and intensity of infection against age, adjusted for gender and location. Odds ratio represents the increase for each year

	Odds ratio	95% CI	*P*	Adj. odds ratio^a^	95% CI	*P*
EPG	1.18^b^	(1.11–1.26)	< 0.0005	1.18^b^	(1.11–1.26)	< 0.0005
CCA	1.27^c^	(1.09–1.49)	0.003	1.26^c^	(1.07–1.48)	0.005

*Abbreviations*: *CCA* circulating cathodic antigen, *EPG* eggs per gram

^a^Odd ratio adjusted for sex and site

^b^Pooled odds ratio from an ordinal logistic regression of 3 binary outcomes (> 0, > low, > moderate)

^c^Odds ratio from a binary logistic regression

CCA analysis of the urine samples for *S. mansoni* revealed a positive rate of 374/399 (93.7%). Of all positive samples, trace and single positive graded infections were found in 115/374 (31%), double positive in 128/374 (34%) and triple positive in 131/374 (35%). Analysis showed a positive correlation between CCA grading of infection and age (binary regression model, *P* < 0.05), with a pooled odds ratio of 1.26 for higher CCA grading with age (95% CI: 1.07–1.48), adjusted for site and gender.

With regards to urinary schistosomiasis, 399 urine samples were analysed. There were 13/399 (3.2%) samples positive for microhaematuria and of these, all were negative for *S. haematobium* eggs.

## Discussion

This cross-sectional study demonstrates an alarmingly high endemicity of *S. mansoni* infection in the Marolambo District. *Schistosoma mansoni* eggs were detected in faecal samples from 73.6% of children and 93.7% of children had positive CCA tests. This high prevalence could be explained by the lack of PC in these communities since 2008. We found that age had a strong positive correlation with both prevalence and infection intensity as demonstrated by both Kato-Katz and CCA. There were no differences in either prevalence or infection intensity between villages or genders.

As expected from its known distribution, *S. haematobium* was not endemic. Although 13 samples (3.2%) were positive for microhaematuria, urine filtration of these samples revealed no infection with *S. haematobium.* Microhaematuria may be attributed to a number of alternative possible causes such as bacterial infection of the urinary tract and inflammation, renal disease, exercise, trauma and menses.

The discrepancy in detection rates of *S. mansoni* between Kato-Katz and CCA seen here is well recognised; sensitivity of single smears of Kato-Katz is lower than that of CCA analysis. The low sensitivity associated with Kato-Katz may be due to variations in the distribution of schistosome eggs between stool samples and there may be reduced detection rates in either light or recently acquired infections [29–31]. One study limitation is that single smears were taken for Kato-Katz analysis and it is possible that low intensity infections were not identified, though these are likely to have been picked up by CCA.

The high prevalence values seen in Marolambo are comparable to other focal prevalence surveys in Tanzania (64.3%) [16], Western Kenya (60.5%) [32] and the Democratic Republic of the Congo (82.7%) [33]. Previously, similar remote-area expeditions have been carried out in Madagascar by Howarth et al. [26] who found an overall prevalence of 69% of *S. haematobium* infection across two schools in the Ankilivalo District of Western Madagascar. They found a marked increase in prevalence from 13% in 1971 to 74% in 1988 in one school where there had been the introduction of a new irrigation system [26]. More recently, a survey was carried out in 2015 across randomly selected sites untreated for at least five years in western Madagascar. The study revealed *S. haematobium* infection prevalence rates higher than 90% in some locations, and higher than 80% prevalence of *S. mansoni* infection in one location (Mitia-Est) [25]. Other recent studies in central Madagascar in the Amoron’i Mania region have found *S. mansoni* infection rates of 77.1% in schools in the Ampasina village, Ambositra [27] and 68.3% in the Tetikanana village, Ambatofinandrahana [34]. Our results from SAC in Marolambo demonstrate that schistosomiasis is hyperendemic in another untreated population in Madagascar and provides evidence for the need to include Marolambo in the national NTD programme for PC.

The extremely high prevalence rates found in our study may explain the non-significant differences between villages. Small variations could represent access to adequate water, sanitation and hygiene (WASH) facilities in schools, as well as social and cultural sanitation and hygiene practices [25, 35]. Limited access to WASH facilities have been revealed in western Madagascar (75% of schools had a WASH score < 3); while no significant correlations were found between WASH status and schistosomiasis prevalence or intensity, this may be due to an overall poor degree of adequate WASH facilities [25].

In our study, whilst there was no significant difference between sexes, prevalence and infection intensity increased with age with both Kato-Katz and CCA, in keeping with the literature [36]. This is unsurprising given the absence of treatment with PZQ since 2008. By downwardly extrapolating from the odds ratio of epg values and CCA prevalence, pre-school ages (less than five years of age) are also likely to have schistosomiasis, with moderate intensities expected to be seen in three and four year olds and low intensity infections in approximately 50% of two year olds. This indicates that testing and treating pre-school children should be considered in Marolambo [14]. When made palatable for very young children, PZQ has been shown to be safe, well tolerated and effective [37]. However, the WHO currently advises against MDA treatment pre-school children due to the lack of paediatric PZQ formulations. Pre-school children should instead be treated on an individual basis by child-health services, where their weight can be monitored and nutrient supplements provided [21, 38].

Heavy infection intensities, determined by epg values exceeding 400, were common in the study population. Children with higher *S. mansoni* egg counts suffer from a higher burden of disease, evidenced by presence of faecal occult blood, faecal calprotectin, worsening degrees of anaemia, fitness levels, growth rates and liver fibrosis [8–12, 14, 39, 40].

Schistosomiasis associated morbidity and mortality can be improved by implementing WHO guidance with regular MDA [21]. Whilst PC is vital to control morbidity, adjunctive approaches should be considered to reduce the prevalence of heavy infection intensities found in Marolambo. These approaches should include health education and encouragement of behavioural change, the provision of safe water, sanitation and hygiene, and snail control strategies [18].

The project had some limitations. Due to time constraints we were unable to screen for presence of soil-transmitted helminths. Owing to errors with staining we were unable to analyse faecal samples from one school in Marolambo, so these samples could not be included in our study. The study sites (schools) were purposively selected due to the logistics of reaching each school, proximity to the Nosivolo River and the fact that the disease had not been studied here before or the population treated since 2008. Although our results demonstrate high endemicity amongst these sites they do not represent the entire Marolambo district.

Non-school-attending SAC were not included in this study. Some studies have shown that non-attenders have similar or sometimes higher prevalence rates of infection when compared to attending school children, and higher infection intensities have been associated with poor attendance rates [25, 41–45]. Although current WHO guidance suggests MDA based in schools as the optimal delivery location to treat SAC, efforts should also be made to treat those who are not enrolled in schools [21, 46].

## Conclusions

Intestinal schistosomiasis is hyperendemic among SAC in the remote Marolambo district of Madagascar where it is likely to have a considerable public health impact. Prevalence rates of greater than 50% were found in each village highlighting the vital need for annual PZQ treatment in accordance with WHO guidelines. These results have been shared with the Madagascar MOH to guide the national MOH-led NTD-programme. In addition to PC, improving health education, as well as access to safe water, sanitation and hygiene would be suitable complementary measures to reduce the prevalence and burden of schistosomiasis.

## Abbreviations

CCA: Circulating cathodic antigen
EPG: Eggs per gram
MDA: Mass drug administration
MOH: Ministry of Health
PC: Preventative chemotherapy
PZQ: Praziquantel
SAC: School-aged children
WHO: World Health Organization
